# Modified Tension Band Wiring Technique for Patella Fractures: A Case Report

**DOI:** 10.7759/cureus.82077

**Published:** 2025-04-11

**Authors:** Harshal Hurkat, Clevio Desouza

**Affiliations:** 1 Orthopaedics, Jupiter Hospital, Indore, IND; 2 Orthopaedics, Saifee Hospital, Mumbai, IND

**Keywords:** biomechanical strength, early mobilization, modified technique, patella fracture, tension band wiring

## Abstract

Patellar fractures are common injuries that compromise the extensor mechanism of the knee. Tension band wiring (TBW) is a widely accepted surgical technique for simple mid-pole fractures, but is associated with complications like wire migration and breakage. This case report presents a modified TBW technique designed to improve biomechanical stability and reduce complications. A 51-year-old female patient presented with right knee pain and swelling following a fall. Radiographs confirmed a transverse patella fracture. Surgical fixation was performed using a modified TBW technique involving triple-bundle wire stitching to enhance stability and prevent migration. Postoperative rehabilitation included early mobilization with continuous passive motion and gradual weight-bearing. Follow-up at three months demonstrated fracture union and excellent knee function without complications. The modified TBW technique demonstrated stability and allowed early functional recovery without implant-related complications. This technique may be considered for broader clinical application.

## Introduction

The patella, the largest sesamoid bone in the human body, plays a crucial role in the knee's extensor mechanism. Patellar fractures comprise approximately 1% of all skeletal injuries in adults and often result from direct trauma or forceful quadriceps contraction [1]. These injuries frequently disrupt the extensor mechanism and typically require surgical fixation to restore both patellar congruity and knee extension [2,3].

Comminuted fractures of the patella, classified as AO Foundation/Orthopaedic Trauma Association (AO/OTA) type 34-C3, present significant challenges in achieving anatomical reduction and stable internal fixation due to multiple fracture fragments and disrupted soft tissue attachments. The AO Surgery Reference recommends cerclage compression wiring as a standard technique in such cases to achieve circumferential stabilization [4]. However, this approach can be associated with complications such as soft-tissue irritation and implant-related discomfort. In contrast to simple transverse fractures, where tension band wiring (TBW) remains the gold standard, comminuted patterns are less amenable to traditional TBW techniques due to their inherent instability and risk of fixation failure [5]. 

This case report describes a modified TBW technique tailored for AO/OTA 34-C3 fractures, aiming to enhance fixation strength, achieve better fragment control, and minimize complications such as hardware migration, prominence, and failure. By addressing the unique biomechanical demands of comminuted patellar fractures, this technique seeks to improve postoperative outcomes in complex fracture scenarios.

## Case presentation

**Figure 1:**
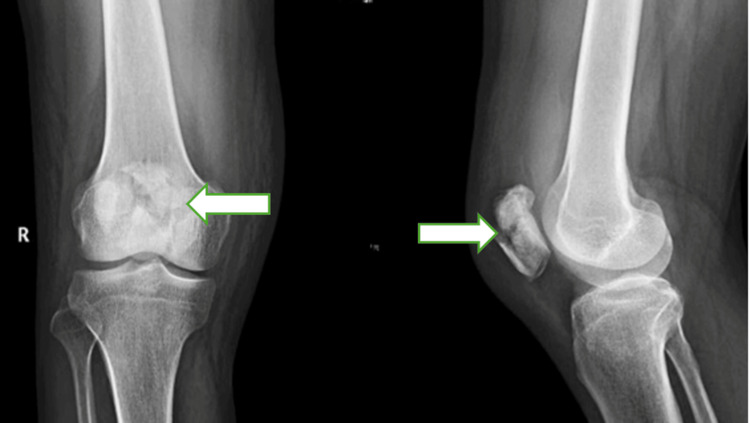
Preoperative radiograph (anteroposterior and lateral views) showing comminuted patella fracture (AO/OTA 34-C3) with multiple displaced fragments.

A 51-year-old female patient presented with acute right knee pain, swelling, and inability to bear weight after a mechanical fall at home while descending stairs, resulting in a direct blow to the anterior aspect of her flexed knee. Clinical examination revealed prepatellar swelling, a palpable bony gap over the patella, and inability to perform active knee extension, suggestive of extensor mechanism disruption. Radiographs demonstrated a comminuted patella fracture (AO/OTA 34-C3) with multiple displaced fragments (Figure 1).

Surgical intervention was planned under spinal anesthesia, with the patient positioned supine and a thigh tourniquet applied. A longitudinal midline anterior incision was made to expose the fracture. Hematoma evacuation and debridement were performed to visualize the articular surface. Fracture fragments were mobilized and reduced using pointed reduction forceps under direct visualization.

Surgical technique

**Figure 2:**
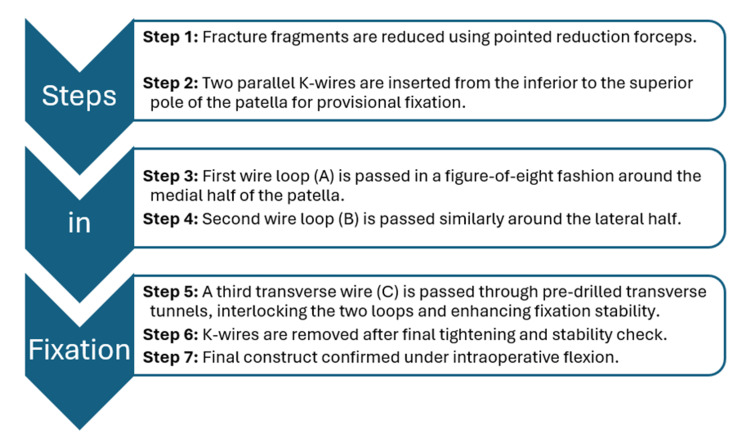
Surgical Steps for the Modified Tension Band Wiring Technique

The surgical technique is described in Figure 2.

**Figure 3:**
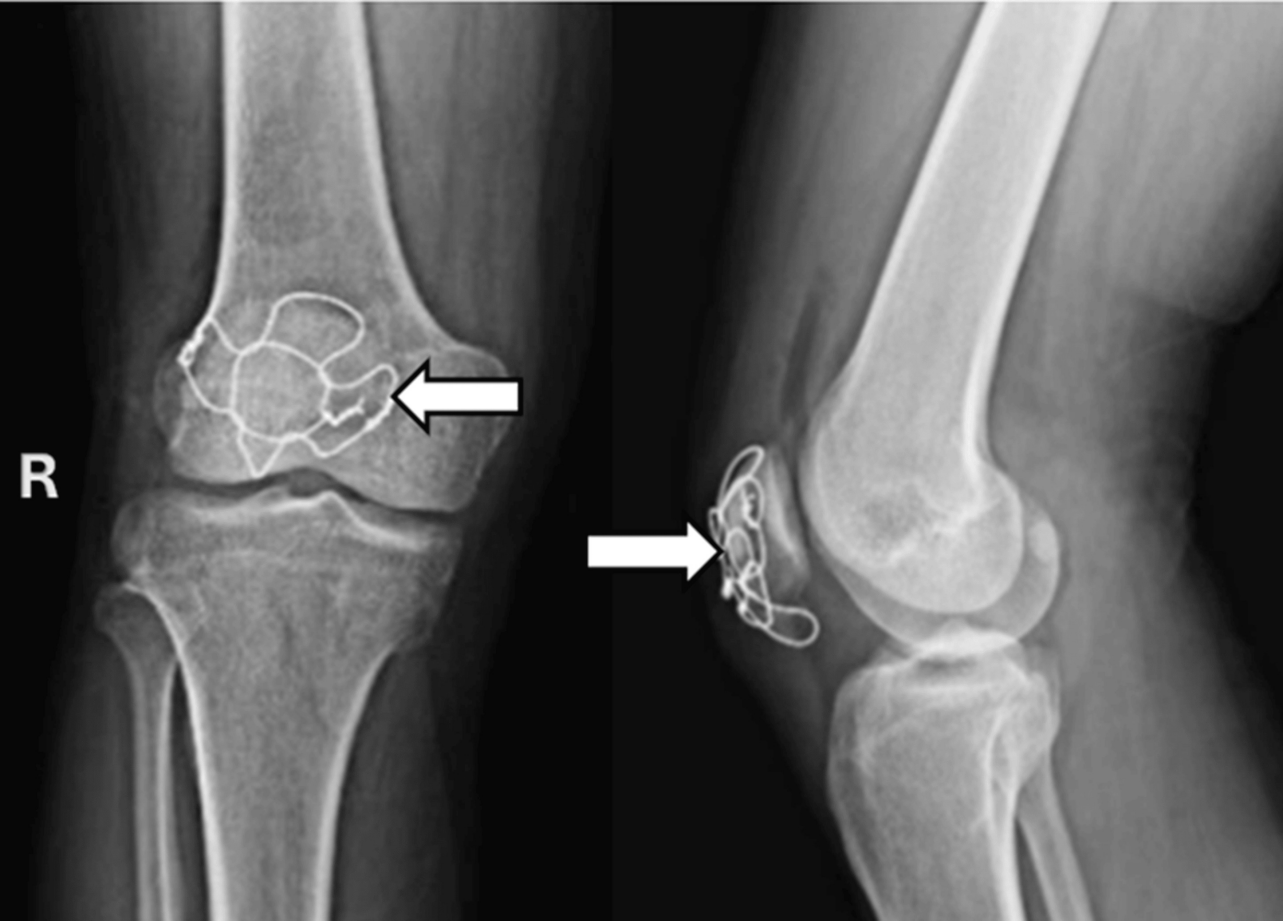
Postoperative radiograph showing patella fixation with the modified triple fixation technique

Firstly, temporary fixation was achieved using two parallel 1.6 mm K-wires placed longitudinally from the inferior to the superior pole. Then, a modified triple-bundle stainless steel wire construct was applied: Two stainless steel wires of 1.5 mm were passed in a figure-of-eight fashion around each half of the patella to secure major fragments. A third wire was passed transversely through pre-drilled holes, interlocking the first two and enhancing the overall stability. Once definitive fixation was confirmed, the temporary K-wires were removed. The construct’s stability was verified through 90 degrees of knee flexion. The retinaculum was repaired, hemostasis was achieved, and the wound closed in layers (Figure 3).

**Figure 4:**
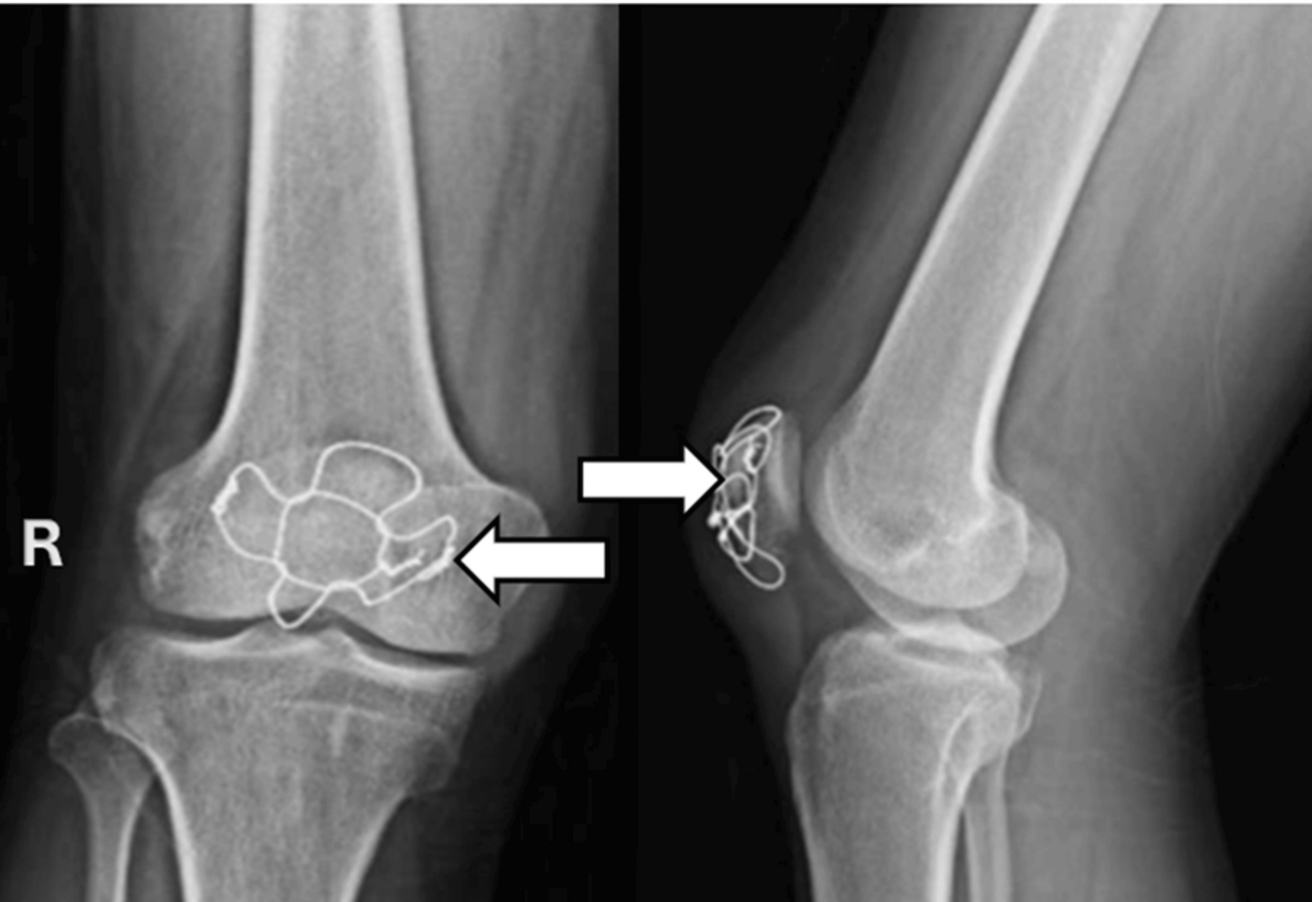
Radiograph (anteroposterior and lateral views) at three months showing union.

Postoperatively, a long knee brace was applied to maintain the knee in full extension and protect the repair. Continuous passive motion (CPM) and isometric quadriceps exercises were initiated on the first postoperative day to prevent stiffness and promote early mobilization. The patient was allowed partial weight-bearing with crutches for the first four weeks. From the fifth week onward, she progressed to full weight-bearing as tolerated. At the three-month follow-up, radiographs confirmed complete fracture union (Figure 4), and the patient had regained full, pain-free range of motion with independent ambulation.

## Discussion

Traditional TBW techniques are widely used for patellar fractures but are associated with complications such as K-wire migration, wire breakage, and hardware irritation [6,7]. Although alternative fixation methods like circumferential cerclage wiring and low-profile plating have been introduced to overcome these issues, they often entail increased surgical complexity, cost, and soft tissue dissection [8,9].

In this case, we employed a modified triple-bundle stainless steel wiring technique, designed to enhance the biomechanical strength of the construct. The configuration provides built-in redundancy-ensuring that if one wire fails, the remaining components preserve fixation integrity. Moreover, positioning the wires beneath the retinaculum and in close contact with the K-wire trajectory minimizes the risk of migration and symptomatic hardware. The stable construct allowed for early mobilization, which is crucial for preventing joint stiffness, promoting synovial circulation, and expediting functional recovery.

The AO Surgery Reference recommends cerclage compression wiring as a standard approach for managing AO/OTA 34-C3 multifragmentary patellar fractures due to its ability to provide circumferential compression and stabilize multiple fragments [4]. However, this technique may still be prone to issues such as wire loosening, inadequate fixation in osteoporotic bone, and difficulty in achieving uniform compression across irregularly shaped fragments. Our modified triple-bundle wiring technique offers several distinct advantages. First, by dividing the construct into medial and lateral figure-of-eight loops with an interlocking transverse third wire, the system provides multi-directional stability and segmental redundancy, reducing the risk of catastrophic failure if one wire loosens or breaks. Second, the targeted anchorage of wires around each half of the patella allows for individualized fragment compression rather than relying solely on circumferential tension. Additionally, by placing the construct beneath tendinous structures, wire migration is minimized. This method simplifies the procedure without requiring complex instrumentation or high-cost implants, making it particularly useful in resource-constrained settings. While both methods aim to restore the extensor mechanism and articular congruity, our approach prioritizes low-profile, high-stability fixation that facilitates early mobilization and may offer superior comfort and outcomes in selected patients. 

While this report describes a single case, the favorable radiological and functional outcomes suggest that this technique could be a viable alternative in managing comminuted patellar fractures, particularly AO/OTA 34-C3 types. However, further prospective studies with larger sample sizes and biomechanical validation are necessary to confirm its efficacy, reproducibility, and long-term safety.

## Conclusions

The modified triple-bundle TBW technique provided stable fixation, enabled early mobilization, and led to favorable functional outcomes in a comminuted patellar fracture (AO/OTA 34-C3) without implant-related complications. This case highlights the practical advantages of enhancing traditional TBW with additional wire redundancy, particularly in comminuted fractures where fragment stability is difficult to achieve. By distributing forces across multiple wire loops and minimizing implant prominence, the technique theoretically reduces the risk of wire migration or failure, though this requires validation through larger biomechanical and clinical studies. Its cost-effectiveness and ease of application make it an appealing option for orthopedic surgeons, especially in resource-limited settings. While limited to a single patient, this case supports further investigation into the utility of this technique for managing complex patellar fractures.
